# Caregiving for dementia: trends pre-post onset and predictive factors of family caregiving (2002–2018)

**DOI:** 10.1093/haschl/qxae020

**Published:** 2024-02-16

**Authors:** Bailey C Ingraham, Douglas Barthold, Paul Fishman, Norma B Coe

**Affiliations:** Department of Health Systems and Population Health, University of Washington, Seattle, WA 98195, United States; The Comparative Health Outcomes, Policy, and Economics (CHOICE) Institute, University of Washington Department of Pharmacy, Seattle, WA 98195, United States; Department of Health Systems and Population Health, University of Washington, Seattle, WA 98195, United States; Department of Medical Ethics and Health Policy, Perelman School of Medicine, University of Pennsylvania, Philadelphia, PA 19104, United States

**Keywords:** dementia, caregiving, incidence

## Abstract

Persons living with Alzheimer's and other related forms of dementia rely heavily on care from family and friends for assistance with daily activities (“family care”), but little is known about care transitions over time. We analyzed data from the Health and Retirement Study to describe caregiving patterns, from 2 years before dementia onset and up to 6 years after. Using sociodemographic data from the interview prior to dementia onset, we determined if there are significant factors that predict receipt of family care at dementia onset. We found that one-third (33%) of people living with dementia were receiving help with daily activities 2 years prior to their first positive dementia screen and this increased to 60% during the first positive screen. Nearly all of those receiving assistance received family care. We found multiple significant predictors of receiving family care at onset, including race, education, access to private health insurance, number of activities of daily living that were difficult, number of chronic conditions, and already receiving help. This demonstrates potential gaps in dementia care, and which subpopulations may benefit most from targeted interventions for household members who do not have adequate caregiving resources or programs that provide additional formal care.

## Introduction

Persons living with Alzheimer's disease and other related forms of dementia (PLWD) often require significant caregiving leading up to and during disease onset.^1^ As dementia progresses, greater help is needed with both instrumental activities of daily living (IADLs), such as grocery shopping and managing prescriptions, as well as functional activities of daily living (ADLs), such as getting dressed and bathing.^1^

Most insurance plans in the United States do not cover support with daily activities; instead, this is often provided through help by family members and friends, who are largely unpaid (referred to here as family caregiving).^2^ Alternatively, some support is provided in long-term-care facilities or from professional caregivers visiting the home (referred to here as formal caregiving),^3^ which typically involves out-of-pocket cost. Some expenses can be paid through long-term-care insurance, although only 1 in 10 adults aged 50 years and older currently have such coverage.^1^ Medicaid or Veterans Affairs benefits also cover some services for those who are eligible. However, securing these services can be a challenge depending on geographical access, the insurance accepted, and the number of beds and personnel at a facility.^1^ These expenses can cause financial strain to the PLWD and their family members supporting them.

The impacts of caregiving are well documented. Caregiving may offer some benefits to the unpaid caregiver such as skill-building, satisfaction in caregiving for another, and remaining close to a loved one needing care.^1,4,5^ However, caregiving is demanding, and providing care for a PLWD can be more demanding than caregiving for persons with other conditions and may result in worse physical and mental health for the caregiver.^1,6^ Quantifying the magnitude of caregiving around onset may offer an opportunity for clinical providers and caregivers to plan to how best meet the care needs. Further, it can provide policymakers with the data needed to forecast demand in order to provide adequate services, whether providing caregiver supports to family members caring for PLWD or ensuring that there are robust networks of formal services available.

The extant research on caregiving for PLWD is primarily based on prevalent disease and little is known about the role of family caregiving before dementia onset and as the condition progresses.^7-9^ To our knowledge, only 2 previous studies have examined caregiving and caregiver characteristics longitudinally, and they found that numbers of caregivers and hours of caregiving increased from onset to 8 years post-onset.^10,11^ We extend this research to include the pre-onset period to examine how these critical caregiving patterns evolve over the course of dementia onset. We summarize characteristics of PLWD and their caregivers to describe caregiving that PLWD receive before the onset of dementia and up to 6 years post-onset. We also predict receipt of family care during the onset interview based on information from the interview prior to dementia onset.

## Data and methods

### Data sources

We used data from the Health and Retirement Study (HRS), a biennial longitudinal survey of approximately 20 000 older adults and their spouses conducted since 1992.^12^ The analytic dataset was formed with multiple components, including the following: the RAND HRS Longitudinal file,^13^ the Langa-Weir Classification of Cognitive Function,^14^ the HRS core and exit interview data from the Functional Limitations, ADL/IADL, and Helper modules. We combined data from the first interview at which the respondent screened positive for dementia (interview 1) with information from the immediate prior interview and up to 3 interviews after the first positive interview. The HRS has a robust process for including proxy responses if respondents have limited capacity to answer questions due to health or cognitive issues and those proxy responses were included in this study.^12^ This study uses data from 2002 to 2018, during which the survey questions regarding caregiver relationship had consistent response options. For respondents who died, we used information from the exit interview to report on nursing home status, receiving help for ADLs and IADLs, caregiving frequency, and caregiver attributes.

### Study population

Survey participants were included in the sample if they had their first positive dementia screen between 2004 and 2018 based on the Langa-Wier Classification.^14^ A participant's first positive dementia screening was defined as the first interview where they were classified as having “Probable Dementia” and they had a prior interview with a nonmissing cognitive score and a confirmed classification of either “Cognitively Normal” or “Cognitively Impaired, No Dementia.” Participants with a positive dementia screen were excluded if they were younger than 50 years old, or if their spouse also had a positive screen for dementia during that interview or during a prior interview.

Previous research^15^ has raised the concern that test results of cognitive function may fluctuate, and a single positive finding is insufficient to confirm dementia status. To reduce this concern, the study population was further restricted to those who did not have a screen of “Cognitively Normal” in all 3 interviews following the initial positive screen. However, participants did remain in the sample if a post-interview screening had a “Cognitively Impaired, No Dementia” screen or did not have complete data during the 3 post-interviews due to death, nonresponse, or the end of the study period. The final sample included 2706 HRS participants with dementia who were living in the community or a nursing home at the time of the interview.

### Outcomes

Recording caregiver information in the HRS is conditional on the respondent or their proxy reporting having difficulty with an ADL or IADL and reporting that they receive help for at least 1 ADL or IADL. Activities of daily living and IADLs are a common measure that indicate physical and cognitive limitations of a person's ability and are a strong predictor of the need for both family and formal caregiving.^16^ Activities of daily living include bathing, dressing, walking across the room, getting in and out of bed, and using the toilet; IADLs include using the telephone, taking medications, managing money, shopping for groceries, and preparing hot meals. Respondents report helpers for each type of activity (ADLs, IADLs, and money management) separately. Participants could report multiple helpers and a variety of characteristics for each helper, such as the relationship of the helper, the number of hours they help, and whether they are paid. Indicators of receiving help for each of these activities are described over time in our analyses to inform what type of activities participants need help with as dementia progresses.

The primary outcome for receiving family care was defined as the respondent or their proxy reporting that (1) they had difficulty with at least 1 ADL or IADL, (2) they received help for an ADL or IADL, and (3) they report information on at least 1 helper whose relationship was a family member or friend, rather than paid or professional care. Appendix S1 outlines how the helper relationship variable was constructed for this study.

In addition to caregiver relationship and family care receipt, we examined the number of ADLs and IADLs that are difficult and frequency of help with ADLs and IADLs over time. We also summarize the type of care received (none, family only, formal only, and a combination of family and formal care) along with care intensity measured by hours provided per month. Descriptive statistics of hours only included PLWD who had complete hourly data for each helper reported. These specific statistics only represent 42%–77% of the population receiving help at each interview.

Nursing homes are another setting where PLWD can receive help with ADLs and IADLS, either as a substitute or a complement to family care.^8^ We therefore also summarized nursing home utilization using data from the RAND Longitudinal file to describe where participants received caregiving. Other characteristics describing persons living with dementia were pulled directly from the HRS RAND Longitudinal file, including sociodemographic information, working status and income, chronic conditions, mortality, functional limitations, and some measures of social resources available.

### Statistical approach

We examined sociodemographic characteristics for PLWD, their utilization of nursing home facilities, and characteristics of the care they received before and after their first positive screen. Further, we modeled the probability of receiving family care during the onset interview as a function of information from the previous interview using logistic regression. This model was used to predict receiving family care based on the following: age, gender, race, ethnicity, educational level, marital status, income and wealth quartiles, working status, number of chronic conditions, health insurance type, long-term-care insurance, life insurance, difficulty with ADLs or IADLS, receiving family care, nursing home status, number of living children, friends nearby (within 10 miles), and receiving help with yard work or chores due to a health issue. All data processing and analyses were conducted in Stata SE 14 with the Margins command used to calculate predicted probabilities.^17^

## Results

**Figure 1.**
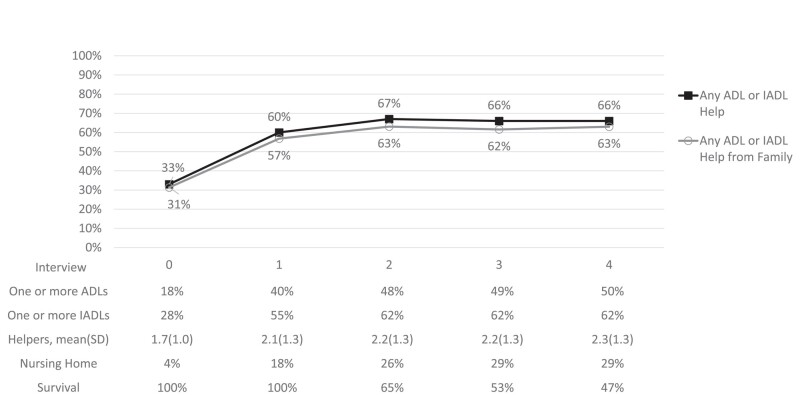
Percentage of the population receiving help for ADLs and IADLs pre- and post-dementia onset. Source: Authors’ analysis of data from the Health and Retirement Study, 2002–2018. Participants could report multiple helpers and a variety of characteristics for each helper, such as the relationship of the helper and whether they are paid. The population includes all PLWD with complete survey or exit interview information, even those who did not report difficulties. Abbreviations: ADL, activity of daily living; IADL, instrumental activity of daily living; PLWD, persons living with dementia; SD, standard deviation.

Figure 1 reports survival and caregiving changes over time, including receiving help for ADLs and IADLs, help from family, nursing home status at time of interview, and mean number of helpers. By the end of the study period only 47% of the cohort were still alive. The biggest increase in receiving help occurred between the interview prior to onset (interview 0) and onset (interview 1), with the proportion of those receiving any help for ADLs and/or IADLs increasing from 33% to 60%. Across the entire study period, nearly all of those receiving help for ADLs or IADLs were receiving some help from at least 1 family member. The proportion of the population in nursing homes increased from 4% prior to onset to 18% at onset, and reached 29% by the 2 last interviews captured.

For those receiving help, the mean and standard deviation (SD) number of helpers was 1.7 (SD = 1.0) prior to onset and slightly higher, 2.1 (SD = 1.3), at the onset interview and slightly higher, 2.3 (SD = 1.3), at the last interview. More details are available in Appendixes S2–S4. Appendix S2 shows caregiving over time broken down by type of activity (ADLs, IADLs, and money help). Appendix S3 is a detailed table describing if help was received for each individual ADL and IADL activity and the primary helper for each type of activity. Appendix S4 has greater detail on PLWD interview response rates, mortality, and nursing home usage.

### Population descriptives

Table 1 summarizes demographic information for the entire population, stratified by receipt of family care during the first positive dementia interview. The average age at the first positive interview was 78 years, and 39.2% of the study population were men. A small percentage (5.9%) were still working. Most (72.2%) of the study population were White, 20.6% were Black/African American, and a small percentage (7.2%) were American Indian, Alaskan Native, Asian, or Pacific Islander. Fourteen percent were Hispanic. Twenty-eight percent had more than a high school education, and few (7.5%) reported having no living children.

**Table 1.** Sample demographics by receipt of family care at the onset interview.

Characteristics of PLWD	All PLWD (*n* = 2706)	Not receiving family care (*n* = 1168)	Receiving family care (*n* = 1538)	Difference^a^
Age, mean (SD), y	78.0 (11.0)	75.2 (11.1)	80.0 (10.4)	4.8
Male, *n* (%)	1060 (39.2%)	458 (39.2%)	602 (39.1%)	−0.1%
Married, *n* (%)	1134 (41.9%)	469 (40.2%)	665 (43.2%)	3.1%
White, *n* (%)	1954 (72.2%)	750 (64.2%)	1204 (78.3%)	14.1%
Black/African American, *n* (%)	558 (20.6%)	312 (26.7%)	246 (16.0%)	−10.7%
Other race, *n* (%)	194 (7.2%)	106 (9.1%)	88 (5.7%)	−3.4%
Hispanic, *n* (%)	380 (14.0%)	200 (17.1%)	180 (11.7%)	−5.4%
Less than high school, *n* (%)	1037 (38.3%)	515 (44.1%)	522 (33.9%)	−10.2%
High school or GED, *n* (%)	921 (34.0%)	390 (33.4%)	531 (34.5%)	1.1%
Some college or more, *n* (%)	748 (27.6%)	263 (22.5%)	485 (31.5%)	9.0%
Working for money, *n* (%)	159 (5.9%	133 (11.4%)	26 (1.7%)	−9.7%
Number of chronic conditions, mean (SD)	3.0 (1.6)	2.5 (1.5)	3.4 (1.5)	0.9
Medicare, *n* (%)	2334 (86.3%)	943 (80.7%)	1391 (90.4%)	9.7%
Medicaid, *n* (%)	562 (20.8%)	221 (18.9%)	341 (22.2%)	3.3%
Private health insurance plan, *n* (%)	973 (36.0%)	348 (29.8%)	625 (40.6%)	10.8%
Long-term-care insurance, *n* (%)	261 (9.6%)	76 (6.5%)	185 (12.0%)	5.5%
Life insurance, *n* (%)	1236 (45.7%)	514 (44.0%)	772 (50.2%)	6.2%
Some difficulty with ≥1 ADL, *n* (%)	1365 (50.4%)	222 (19.0%)	1143 (74.3%)	55.3%
Some difficulty with ≥1 IADL, *n* (%)	1639 (60.6%)	165 (14.1%)	1474 (95.8%)	81.7%
No living children, *n* (%)	202 (7.5%)	107 (9.2%)	95 (6.2%)	−3.0%
Proxy, *n* (%)	880 (32.5%)	60 (5.1%)	820 (53.3%)	48.2%

Abbreviations: ADL, activity of daily living; GED, General Educational Development; IADL, instrumental activity of daily living; PLWD, persons living with dementia; SD, standard deviation.

Source: Authors’ analysis of data from the Health and Retirement Study, 2002–2018.

^a^Difference receiving family care minus not receiving family care.

Most reported Medicare and/or a private plan health insurance coverage, and 20.8% self-reported having Medicaid coverage. Most (82%), but not all, of the Medicaid beneficiaries were dual-eligible for Medicare because the study population included PLWD aged 50 years and older. Only 9.6% had long-term-care insurance and nearly one-third had a proxy respondent at the time of their first interview.

Half of respondents reported some difficulty with at least 1 ADL and 60.6% reported some difficulty with at least 1 IADL. High blood pressure and arthritis were the top 2 most reported conditions (>65%). Appendix S5 has greater demographic details, including income, each reported condition, cognition scores, and specific ADLs and IADLs.

Fifty-seven percent of the population reported receiving family care at the onset interview. The majority (92.6%) of those not receiving family care (*n* = 1168) were also not receiving any formal help for ADLs or IADLs, although 19.0% reported difficulty with at least 1 ADL and 14.1% reported difficulty with at least 1 IADL. Those receiving family care were nearly 5 years older, had difficulty with a greater number of IADLs and ADLs, were more likely to be insured, and to identify as non-Hispanic and White.

### Type and intensity of caregiving

**Figure 2.**
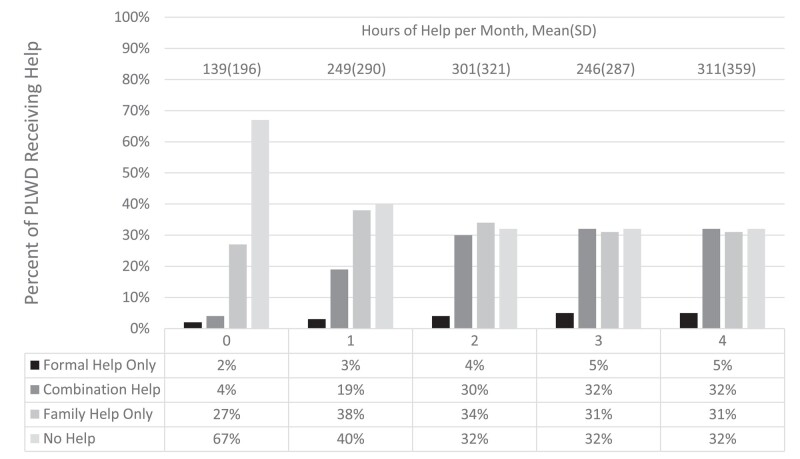
Proportion of PLWD population receiving help pre- and post-dementia onset and number of hours of help received per month pre- and post-dementia onset. Source: Authors’ analysis of data from the Health and Retirement Study, 2002–2018. Summary statistics presented as bars include all PLWD with complete survey or exit interview information, even those who did not report difficulties. Summary statistics on hours only include those who reported at least 1 helper and had complete hourly data on all the helpers they reported. Abbreviations: PLWD, persons living with dementia; SD, standard deviation.

Figure 2 depicts types of care received over the study period with hours of help received for each. Combination help denotes when at least 1 family helper and at least 1 formal helper were reported during the same interview. Over the follow-up period there was large decrease in the proportion of those receiving no help for ADLs or IADLs and a large corresponding increase in those receiving a combination of family and formal care. There was also a modest increase in family help only, and small increase in formal-only care. For hours of care per month, there is a large increase during onset and a modest increase in the first 2 years. In the following years, the mean hours per month varied between 246 and 311. This may be due to a lower sample size for the later interviews, resulting in volatile estimates. Appendix S6 includes more detail on hours by care type received.

### Predicting receipt of family care

Figure 1 shows that, by the first post-onset interview, the suvival rate was 65%, and this decreased to 47% by the third post interview (∼6 years after onset). In post-interviews, those surviving were more likely to have stays in nursing homes and for those stays to be longer. Appendix S4 has more detailed information on mortality, loss to follow-up, and nursing home utilization over time.

There were multiple factors before onset that significantly predicted receiving family care during the onset interview (Table 2). Those receiving help with household chores in the prior interview and those already receiving family care for ADLs and IADLs were more likely to receive family care in the onset interview. White PLWD were more likely (7.3 percentage points) to receive family care during their onset interview than Black/African-American PLWD. Those with higher educational levels and private insurance were more likely to receive family care. A greater number of chronic conditions and number of difficult IADLs reported in the prior interview also predicted greater likelihood of receiving family care during the onset interview. The full regression analysis specification and output are in Appendix S7.

**Table 2.** Regression-generated predicted probabilities of receiving family care during the onset interview based on factors from the prior interview.

Persons living with dementia characteristics prior to onset	Percentage point difference estimate	95% CI	*P*
Race			
White/Caucasian	7.3	[2.5, 12.1]	.003
Other	4.4	[−3.7, 12.6]	.285
Black/African American (reference = 51.7%)	—		
Level of education			
Some college or more	8.5	[3.8, 13.2]	<.001
GED or high school	5.0	[0.8, 9.2]	.021
Less than high school (reference = 52.8%)	—		
Private health insurance?			
Yes	4.4	[0.7, 8.1]	.020
No (reference = 55.0%)	—		
Receiving help with chores or yard work?			
Yes	7.9	[4.0, 11.8]	<.001
No (reference = 54.3%)	—		
Currently receiving family care?			
Yes	19.6	[13.8, 25.4]	<.001
No (reference = 51.8%)	—		
Years of age	0.6	[0.4, 0.8]	<.001
Number of chronic conditions	2.7	[1.5, 3.9]	<.001
Number of IADL limitations	6.6	[3.8, 9.4]	<.001

Abbreviations: ADL, activity of daily living; CI, confidence interval; GED, General Educational Development; IADL, instrumental activity of daily living.

Source: Authors’ analysis of data from the Health and Retirement Study, 2002–2018. For categorical characteristics, like race, the difference estimate refers to the percentage point difference in predicted probability of receiving family care compared with the reference category's rate of receiving family care. For continuous or counted variables, like age, the difference is the percentage point difference in predicted probability associated with a single unit increase, such as 1 year. The logistic regression model adjusted for age, gender, race, ethnicity, educational level, marital status, income and wealth quartiles, working status, number of chronic conditions, health insurance type, long-term-care insurance, life insurance, difficulty with ADLs or IADLS, receiving family care, nursing home status, number of living children, friends nearby (within 10 miles), future help expectations, and receiving help with yard work or chores. For odds ratios and complete regression output, see Appendix S7.

## Discussion

We found that one-third of PLWD were receiving help with daily activities prior to their first positive dementia screen; this increased to 60% during the first positive screen and persisted in subsequent years. Nearly all of those receiving help were receiving care from family members, with nearly one-third receiving both family and formal help with ADLs or IADLs. These results demonstrate the large and growing demand for family caregiving, and that support services to help caregivers, like those provided in Medicare's new dementia care model (GUIDE [Guiding an Improved Dementia Experience]) covering respite care and family caregiver training, will also be in high demand.^18^

These estimates can potentially contribute to prediction models for the national demand for caregiver support services and their costs, which can support efforts like the RAISE Family Caregivers Act to develop a national family caregiving strategy. One of the Act's products, an inventory of federal programs, along with the National Academy for State Health Policy's Roadmap for States, demonstrates that there is a growing network of caregiver support systems in the United States, but access is limited and varies state to state.^19,20^ Future state-level studies examining the relationship between existing demand for caregiving and support received would inform these agencies if they are reaching their objectives.

This study expands the literature of caregiving trends for PLWD by examining the critical point before dementia onset in addition to the years after, giving insight into how caregiving evolves over time. Estimates of family caregiving rates after onset from our study are slightly lower than those reported in previous studies. This is likely due to our more inclusive study population (14% were between the age of 50 and 64) and our longitudinal study design.^7,10^

We also examined the relationship between characteristics of PLWD prior to dementia onset and receipt of family caregiving after onset. Higher education, along with having more comorbidities and more difficulties with IADLs, predicted higher rates of caregiving. We also found that White PLWD were more likely than Black PLWD to receive any family caregiving. This result differs from that of Friedman et al,^7^ who found that Black PLWD were more likely to receive intense family caregiving, defined as more than 200 hours per month.^11^ The difference in results is likely caused by the definition of family care (receiving any family care vs intense family care). This finding provides important information about potential gaps in dementia care, and which subpopulations may benefit most from targeted interventions for household members who do not have adequate caregiving resources or programs that provide additional formal care.^11^

### Strengths and limitations

Due to our study's focus on time relative to onset, we did not use HRS survey weights and therefore our findings are not nationally representative. The HRS data are highly reliant on self-report and proxy reports, and the switch from self-report to proxy likely co-occurs with cognitive decline. Because of this co-occurrence, there may be extra variation in reporting around our time of predictive interest.

Our analysis is limited to the experience of HRS respondents who reported difficulties with ADLs and IADLs. These are common measures that indicate physical and cognitive limitations and are a strong predictor of the need for both family and formal caregiving. However, these are not the only limitations that may necessitate caregiving, and caregiving may be needed for reasons not related to limitations, such as care coordination and emotional support.^9^ Therefore, our caregiving measures likely underestimate prevalence.

To counter potential biases from household correlation, the population used for this study excludes PLWD who have a positive screen after their spouse previously screened positive. Therefore, this study may be slightly overestimating spousal caregiving for the larger PLWD population.

## Conclusion

Our study describes the way that PLWD receive care for ADLs and IADLs, with the goal of informing both policy and research on how best to address the needs of the growing number of older Americans with dementia and their families. To fully assess the caregiving challenges faced by PLWD and their families, more research and greater investment are necessary to improve the data resources relevant to these questions. Future longitudinal research using representative sampling should develop predictive models that inform the relationship between dementia severity and caregiving gaps over time, allowing policymakers to monitor existing caregiving support efforts and pinpoint new opportunities for effective intervention.

## Supplementary Material

qxae020_Supplementary_Data
