# Supplementary material for: Caregiving for dementia: trends pre-post onset and predictive factors of family caregiving (2002–2018)
Source: Health Aff Sch. 2024 Feb 16;2(3):qxae020. doi: 10.1093/haschl/qxae020 (PMC10986258; doi:10.1093/haschl/qxae020)
Supplement: qxae020_Supplementary_Data [file qxae020_supplementary_data.zip › Full Appendix_revision_clean.pdf]

Appendix 1: Map of Helper Relationship Responses to Helper Categories (Bolded rows indicate formal care)

Value	Description	Helper Type Category	Family vs Formal Care
**2**	Spouse or Partner	Spouse	Family Care
**3**	Son	Child	Family Care
**4**	Stepson	Child	Family Care
**5**	Spouse or Partner of Daughter	Child	Family Care
**6**	Daughter	Child	Family Care
**7**	Stepdaughter	Child	Family Care
**8**	Spouse or Partner of Son	Child	Family Care
**9**	Grandchild	Other Family	Family Care
**10**	Father	Other Family	Family Care
**11**	Father-in-law	Other Family	Family Care
**12**	Mother	Other Family	Family Care
**13**	Mother-in-law	Other Family	Family Care
**14**	Parent	Other Family	Family Care
**15**	Brother	Other Family	Family Care
**16**	Brother of Spouse of Partner	Other Family	Family Care
**17**	Sister	Other Family	Family Care
**18**	Sister of Spouse or Partner	Other Family	Family Care
**19**	Other Relative	Other Family	Family Care
**20**	Other Individual	Other Family	Family Care
**21**	**Organization**	**Formal Care**	**Formal Care**
**22**	**Employee of Facility (for those in a nursing home)**	**Formal Care**	**Formal Care**
**23**	**Paid Helper**	**Formal Care**	**Formal Care**
**24**	**Professional**	**Formal Care**	**Formal Care**
**25**	**Professional (specify)**	**Formal Care**	**Formal Care**
**26**	Late Spouse or Partner	Other Family	Family Care
**27**	Former Spouse or Partner	Other Family	Family Care
**28**	Unlisted Child or Child-in-law	Child	Family Care
**29**	Not Proxy Interview	n/a	n/a
**30**	Former Step-Child	Child	Family Care
**31**	Former Child-in-law	Child	Family Care
**32**	Relationship Unknown	n/a	n/a
**33**	Spouse or Partner of Grandchild	Other Family	Family Care
**34**	Not a Sibling	n/a	n/a
**90**	Other type of Child	Child	Family Care

**91**	Other type of Child-in-law	Child	Family Care
**98**	Don’t Know	n/a	n/a
**99**	Refused	n/a	n/a

***Respondents could report up to 7 helpers for ADL care, 6 helpers for IADL (sans money help) care, and 2 helpers for money management. If they reported at least 1 Family Care Helper and at least 1 Formal Care Helper, then they received combination care (Figure 2).**

APPENDIX 2: Proportion of PLWD Population Receiving Help (Row A) and Number of Caregivers/Helpers (Row B) for ADLs and IADLs Pre and Post Dementia Onset

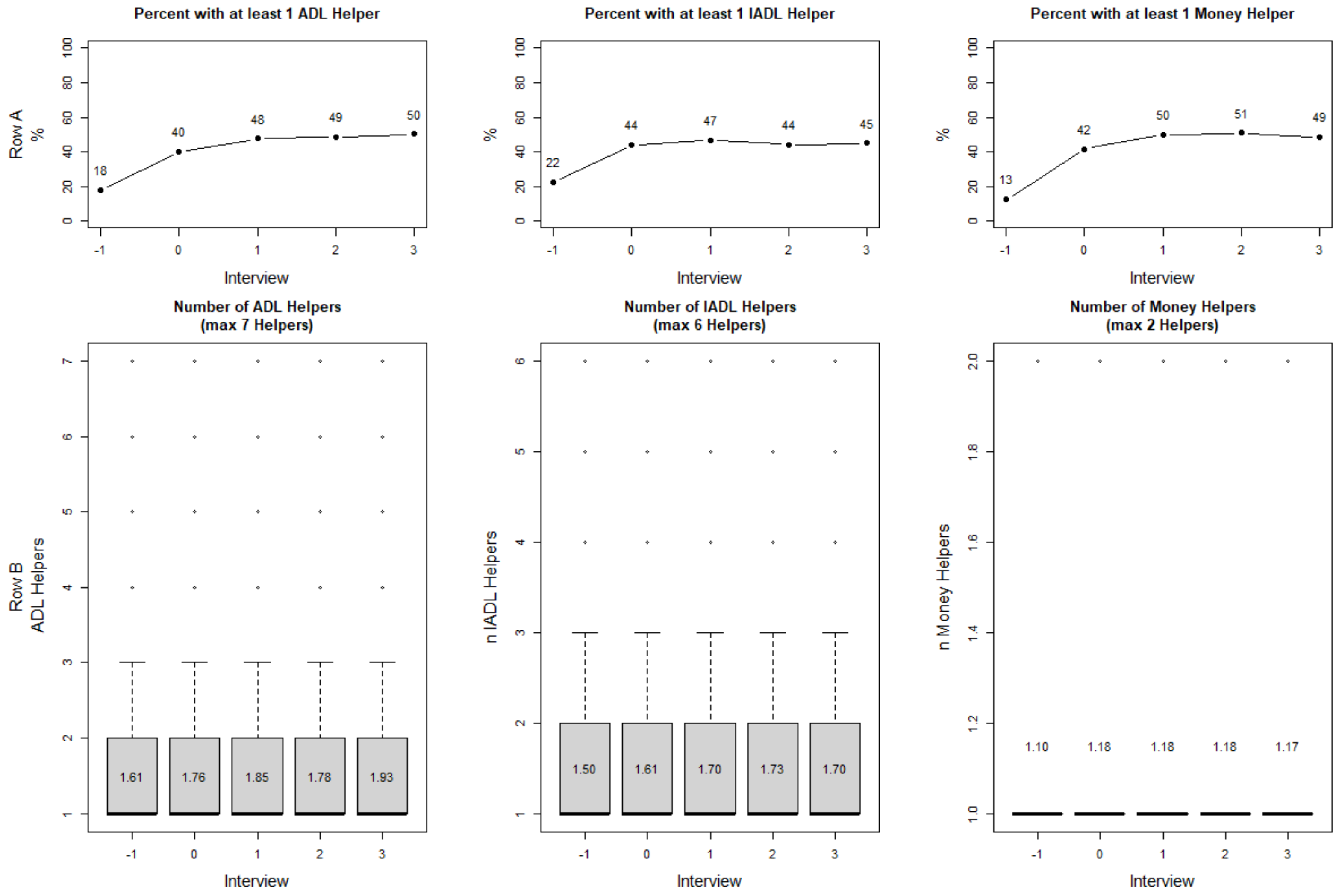

SOURCE [Authors’ analysis of data from the Health and Retirement Study, 2002-2018.] NOTES [PLWD-Persons Living with Dementia; ADLs-Activities of Daily Living; IADLs-Instrumental Activities of Daily Living. The HRS reports helpers for money separately from the other IADLs and this information cannot be combined without double counting. Row A summary statistics presented in the top half include all PLWD with complete survey or exit interview information, even those that did not report difficulties. Row B summary statistics presented in the bottom half only include those that reported at least 1 helper for that activity, whether they be family or formal, paid or not paid. Due to the format of the HRS interview, helper information for money management is reported separately from the rest of the IADLs].

APPENDIX 3: Table of Care Needs and Caregiver Descriptives Pre and Post Dementia Onset

	Interview 0 (-2 years)		Interview 1 (Onset)		Post Interview 1 (+2 years)		Post Interview 2 (+4 years)		Post Interview 3 (+ 6 years)	
Receipt of Care*	n=2706		n=2706		n =1762		n=1058		n=617	
No Help with ADLs or ADLs, n %	1812	67.0%	1082	40.0%	587	33.3%	359	33.9%	207	33.5%
Any Help with ADL or IADL	894	33.0%	1624	60.0%	1175	66.7%	699	66.1%	410	66.5%
Any Family Care	846	31.3%	1538	56.8%	1112	63.1%	652	61.6%	389	63.0%
ADLs Get Help, n %										
At Least 1 ADL	481	17.8%	1082	40.0%	839	47.6%	516	48.8%	310	50.2%
Bathing	300	11.1%	851	31.4%	562	31.9%	342	32.3%	228	37.0%
Eating	111	4.1%	425	15.7%	286	16.2%	175	16.5%	131	21.2%
Dressing	293	10.8%	802	29.6%	543	30.8%	321	30.3%	208	33.7%
Walking Across the Room	180	6.7%	582	21.5%	366	20.8%	216	20.4%	156	25.3%
In/Out Bed	179	6.6%	541	20.0%	362	20.5%	228	21.6%	152	24.6%
Toilet	104	3.8%	433	16.0%	271	15.4%	191	18.1%	132	21.4%
Count, Get Help for 0 ADL	2225	82.2%	1624	60.0%	1065	60.4%	641	60.6%	349	56.6%
Count, Get Help for 1 ADL	203	7.5%	273	10.1%	143	8.1%	89	8.4%	47	7.6%
Count, Get Help for 2 ADL	98	3.6%	185	6.8%	118	6.7%	60	5.7%	35	5.7%
Count, Get Help for 3 or more ADL	180	6.7%	624	23.1%	436	24.7%	268	25.3%	186	30.1%
IADLs Get Help, n %										
At Least 1 IADL	744	27.5%	1491	55.1%	1089	61.8%	654	61.8%	381	61.8%

Phone	189	7.0%	675	24.9%	516	29.3%	303	28.6%	171	27.7%
Medications	124	4.6%	457	16.9%	352	20.0%	208	19.7%	107	17.3%
Money	339	12.5%	1129	41.7%	878	49.8%	539	50.9%	300	48.6%
Grocery Shopping	401	14.8%	700	25.9%	447	25.4%	248	23.4%	146	23.7%
Preparing Meals	265	9.8%	610	22.5%	399	22.6%	220	20.8%	133	21.6%
Count, Get Help for 0 IADL	1962	72.5%	1215	44.9%	673	38.2%	404	38.2%	236	38.2%
Count, Get Help for 1 IADL	407	15.0%	493	18.2%	384	21.8%	251	23.7%	151	24.5%
Count, Get Help for 2 IADL	184	6.8%	444	16.4%	320	18.2%	190	18.0%	105	17.0%
Count, Get Help for 3 or more I IADL	153	5.7%	554	20.5%	385	21.9%	213	20.1%	125	20.3%
Primary Helper for ADLs Relationship**	n=	481	n=	1082	n=	839	n=	516	n=	310
Spouse, n %	175	36.4%	286	26.4%	160	19.1%	96	18.6%	47	15.2%
Child, n %	116	24.1%	209	19.3%	145	17.3%	84	16.3%	59	19.0%
Other Family/Individual, n %	72	15.0%	104	9.6%	87	10.4%	46	8.9%	32	10.3%
Paid Organization or Professional, n %	118	24.5%	483	44.6%	447	53.3%	290	56.2%	172	55.5%
Primary Helper for IADLs Relationship**	n=	604	n=	1188	n=	827	n=	468	n=	278
Spouse, n %	255	42.2%	448	37.7%	261	31.6%	133	28.4%	76	27.3%
Child, n %	232	38.4%	396	33.3%	275	33.3%	170	36.3%	112	40.3%
Other Family/Individual, n %	84	13.9%	142	12.0%	126	15.2%	58	12.4%	37	13.3%
Paid Organization or Professional, n %	33	5.5%	202	17.0%	165	20.0%	107	22.9%	53	19.1%
Primary Helper for Money Help Relationship**	n=	339	n=	1129	n=	878	n=	539	n=	300
Spouse, n %	106	22.0%	378	34.9%	234	27.9%	139	26.9%	69	22.3%

Child, n %	187	38.9%	589	54.4%	505	60.2%	310	60.1%	186	60.0%
Other Family/Individual, n %	42	15.0%	134	9.6%	103	10.4%	55	8.9%	32	10.3%
Paid Organization or Professional, n %	4	0.8%	28	2.6%	36	4.3%	35	6.8%	13	4.2%

SOURCE [Authors' analysis of data from the Health and Retirement Study, 2002-2018.]

NOTES [*Denominator includes participants alive and interviewed, and those that passed and exit information was available. **Denominator included the population if they reported receiving help for that type of activity. ADLs-Activities of Daily Living; IADLs-Instrumental Activities of Daily Living. The HRS reports helpers for money separately from the other IADLs and this information cannot be combined without double counting. Participants could report multiple helpers, and a variety of characteristics for each helper, such as the relationship of the helper and whether they are paid].

APPENDIX 4: Table of Mortality and Nursing Home Utilization Pre and Post Dementia Onset

Interview Status, Mortality, n % cohort	Interview 0 (-2 years) n= 2706		Interview 1 (Onset) n= 2706		Post Interview 1 (+ 2 years) n= 2706		Post Interview 2 (+4 years) n= 2706		Post Interview 3 (+ 6 years) n= 2706	
Alive and Responded, alive by end of wave	2706	100.0%	2247	83.0%	1262	46.6%	753	27.8%	453	16.7%
Alive and Responded, but died by end of wave	0	100.0%	459	17.0%	270	10.0%	126	4.7%	73	2.7%
No Response and death during interview wave, exit interview information is used if available	0	0.0%	0	0.0%	230	8.5%	179	6.6%	91	3.4%
No Response, died in previous wave	0	0.0%	0	0.0%	459	17.0%	957	35.4%	1262	46.6%
Alive but no response this wave	0	0.0%	0	0.0%	196	7.2%	151	5.6%	103	3.8%
Self-selected out of study, this wave or prior	0	0.0%	0	0.0%	0	0.0%	14	0.5%	18	0.7%
No Response, End of Study Period	0	0.0%	0	0.0%	289	10.7%	526	19.4%	706	26.1%
Cohort Mortality, n %	0	0.0%	0	0.0%	959	35.4%	1262	46.6%	1426	52.7%
Surviving Population with NH Utilization data	n=	2706	n=	2683	n=	1709	n=	999	n=	580
Any Time in a NH Since Last Interview, n %	264	9.8%	704	26.2%	569	33.3%	349	34.9%	210	36.2%
Currently in a NH as of Interview/at Death, n %	106	3.9%	475	17.7%	437	25.6%	293	29.3%	167	28.8%
Number of Stays in NH since last interview*, n%										
1 Stay	211	79.9%	511	72.6%	446	78.4%	255	73.1%	161	76.7%
2 Stays	30	11.4%	92	13.1%	56	9.8%	48	13.8%	16	7.6%
3 or More Stays	12	4.5%	51	7.2%	28	4.9%	20	5.7%	10	4.8%
Missing	11	4.2%	50	7.1%	39	6.9%	26	7.4%	23	11.0%
Number of Nights in NH since last interview*, n%										
0-90 Nights	144	54.5%	296	42.0%	174	30.6%	76	21.8%	48	22.9%
91-180 Nights	26	9.8%	66	9.4%	31	5.4%	20	5.7%	11	5.2%
181-365 Nights	24	9.1%	73	10.4%	48	8.4%	40	11.5%	14	6.7%
>365 Nights	50	18.9%	218	31.0%	275	48.3%	183	52.4%	118	56.2%
Missing	20	7.6%	51	7.2%	41	7.2%	30	8.6%	19	9.0%

SOURCE [Authors' analysis of data from the Health and Retirement Study, 2002-2018.] NOTES [*Conditional on any time in a nursing home. NH-Nursing Home. Summary statistics presented include all Persons Living with Dementia with complete survey or exit interview information, even those that did not report difficulties].

APPENDIX 5 Table of Detailed Dementia Population Demographics by Receipt of Family Care at Onset Interview

	All PLWD	Not Receiving Family Care	Receiving Family Care	Difference of Group Average
Sociodemographic	n=2706	n=1168	n=1538	Receiving FC - Not Receiving FC
Household Income,				
median [q25, q75]	$20,000 [$11,964, $35,612]	$18,000 [$10,350, $31,994]	$22,071 [$13,098, $38,400]	$4,071
Wealth w/o IRA,				
median [q25, q75]	$65,000 [$1,500, $232,721]	$50,000 [$875, $188,400]	$76,000 [$2,000, $267,000]	$26,000
No Living Children, n %	202 7.5%	107 9.2%	95 6.2%	-3.0%
One Living Child, n %	303 11.2%	140 12.0%	163 10.6%	-1.4%
Two Living Children, n %	619 22.9%	241 20.6%	378 24.6%	3.9%
Three or More Living Children, n %	1527 56.4%	658 56.3%	869 56.5%	0.2%
Health, Functional Limitations, and Social Resources	n=2706	n=1168	n=1538	Receiving FC - Not Receiving FC
High Blood Pressure, n %	1923 71.1%	784 67.1%	1139 74.1%	6.9%
Diabetes, n %	837 30.9%	348 29.8%	489 31.8%	2.0%
Cancer, n %	573 21.2%	196 16.8%	377 24.5%	7.7%
Lung Disease, n %	392 14.5%	131 11.2%	261 17.0%	5.8%
Heart Problems, n %	1056 39.0%	335 28.7%	721 46.9%	18.2%
Stroke, n %	714 26.4%	193 16.5%	521 33.9%	17.4%

Psyche, n %	751 27.8%	224 19.2%	527 34.3%	15.1%
Arthritis, n %	1862 68.8%	704 60.3%	1158 75.3%	15.0%
Cognition Score (0-27)*, mean (SD)	4.8 1.4	5.0 1.3	4.5 1.6	0.4
ADLs Some Difficulty, mean (SD)	1.7 2.2	0.5 1.2	2.7 2.2	2.2
ADLs Some Difficulty, n %				
Bathing	973 36.0%	97 8.3%	876 57.0%	48.7%
Eating	580 21.4%	40 3.4%	540 35.1%	31.7%
Dressing	972 35.9%	120 10.3%	852 55.4%	45.1%
Walking Across the Room	861 31.8%	121 10.4%	740 48.1%	37.8%
In/Out Bed	714 26.4%	94 8.0%	620 40.3%	32.3%
Toilet	630 23.3%	84 7.2%	546 35.5%	28.3%
IADLs Some Difficulty, mean (SD)	1.9 1.9	0.3 0.8	3.2 1.6	2.9
IADLs Some Difficulty, n %				
Phone	910 33.6%	66 5.7%	844 54.9%	49.2%
Medications	802 29.6%	48 4.1%	754 49.0%	44.9%
Money	1209 44.7%	58 5.0%	1151 74.8%	69.9%
Grocery Shopping	1198 44.3%	77 6.6%	1121 72.9%	66.3%
Preparing Meals	1095 40.5%	77 6.6%	1018 66.2%	59.6%
Future Help**, n %	688 25.4%	679 58.1%	9 0.6%	-57.5%
Family Nearby, n %	673 24.9%	241 20.6%	432 28.1%	7.5%

Friends Nearby, n %	1114 41.2%	502 43.0%	612 39.8%	-3.2%
Extra help with yard work/house chores***, n %	986 36.4%	250 21.4%	736 47.9%	26.5%

SOURCE [Authors' analysis of data from the Health and Retirement Study, 2002-2018.] NOTES [*By definition everyone's score during the first positive interview is below 7. **Suppose in the future, you needed help with basic personal care activities like eating or dressing. Do you have relatives or friends [besides your [husband/wife/partner]] who would be willing and able to help you over a long period of time? ***Receive any help with yard work/house chores (not mentioned in ADL/IADL help) because of a health problem. FC-Family Care; PLWD-Persons Living with Dementia; ADLs-Activities of Daily Living; IADLs-Instrumental Activities of Daily Living; SD-Standard Deviation].

APPENDIX 6: Type of Help Received (Row A) and Number of Hours per Month of Help Received (Row B) for ADLs and IADLs Pre and Post Dementia Onset

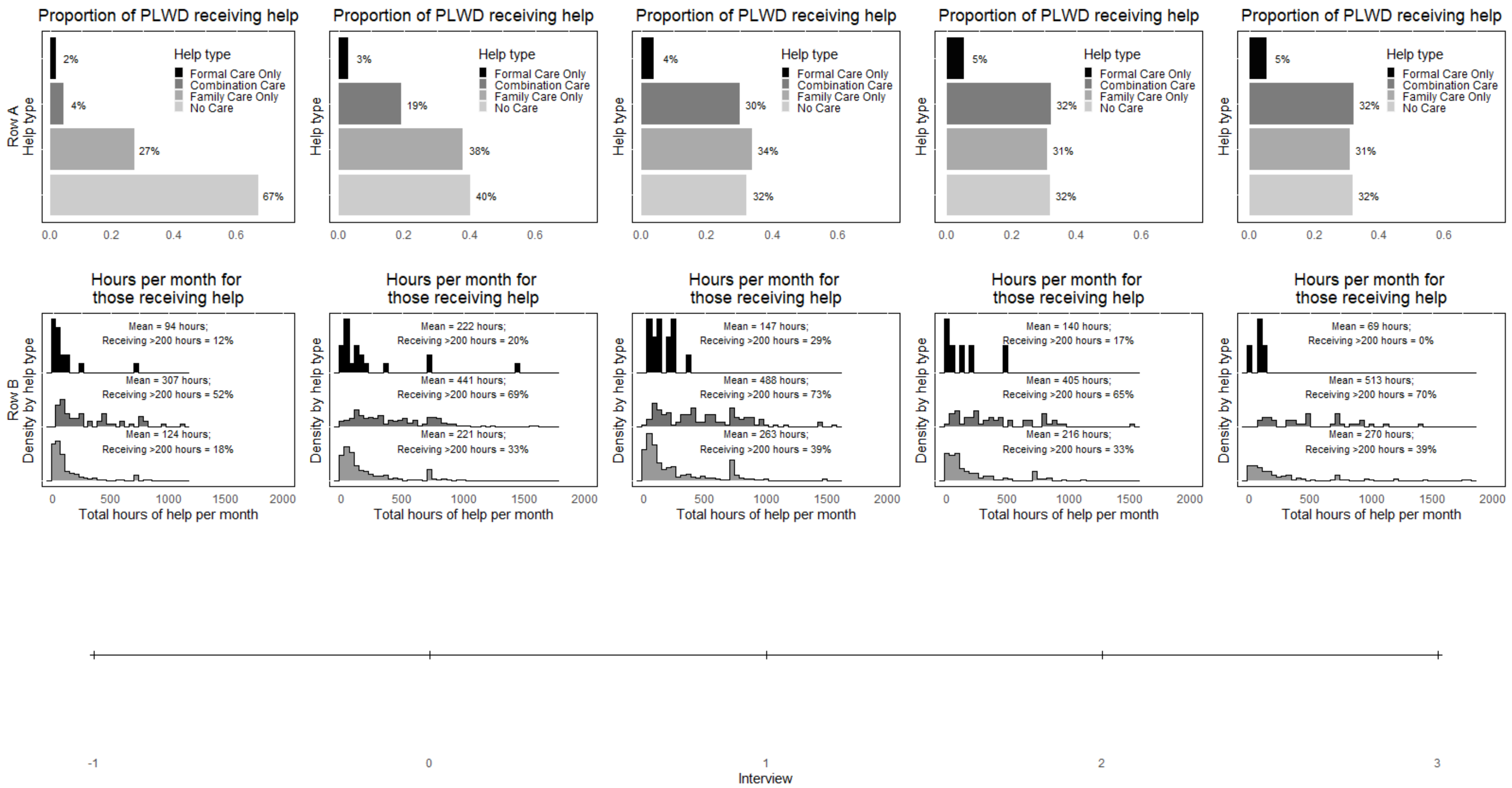

SOURCE [Authors' analysis of data from the Health and Retirement Study, 2002-2018.] NOTES [PLWD-Persons Living with Dementia. Summary statistics presented in the top half include

all PLWD with complete survey or exit interview information, even those that did not report difficulties. Summary statistics presented in the bottom half only include those that reported at least 1 helper and had complete hourly data on all the helpers they reported. Hourly statistics only represent 42-77% of the population receiving help at each interview].

APPENDIX 7: Table of Regression Output Odds Ratios of Receiving Family Care at Onset Interview

	Odds Ratio	Standard Error	p-value	95% Confidence Interval	
Age, years	1.030	0.006	0.000	1.019	1.041
Women, reference Men	0.870	0.086	0.158	0.717	1.056
Married, reference Not Married	1.245	0.144	0.059	0.992	1.561
Race White/Caucasian, reference Black/African American	1.453	0.179	0.002	1.141	1.851
Race Other, reference Black/African American	1.253	0.265	0.286	0.828	1.898
Hispanic, reference Non-Hispanic	0.948	0.145	0.727	0.702	1.280
GED or High School, reference Less than High School	1.288	0.141	0.021	1.039	1.597
Some College or More, reference Less than High School	1.550	0.191	0.000	1.217	1.974
Working for Pay, reference Not Working for Pay	0.751	0.118	0.068	0.553	1.022
2nd Income quartile, reference 1st (Lowest)	0.784	0.112	0.088	0.593	1.037
3rd Income quartile, reference 1st (Lowest)	1.003	0.151	0.983	0.746	1.349
4th Income quartile, reference 1st (Lowest)	0.949	0.158	0.751	0.685	1.314
5th Income quartile, reference 1st (Lowest)	1.107	0.202	0.576	0.774	1.583
2nd Wealth quartile, reference 1st (Lowest)	0.854	0.126	0.284	0.640	1.140
3rd Wealth quartile, reference 1st (Lowest)	1.002	0.154	0.990	0.741	1.354
4th Wealth quartile, reference 1st (Lowest)	0.911	0.150	0.569	0.660	1.257
5th Wealth quartile, reference 1st (Lowest)	0.976	0.175	0.892	0.686	1.388
Extra Chronic Condition	1.149	0.037	0.000	1.079	1.223

Medicare	1.080	0.164	0.611	0.802	1.456
Medicaid	1.037	0.148	0.798	0.784	1.372
Private Health Insurance	1.255	0.122	0.019	1.038	1.519
Long Term Care HI	1.212	0.193	0.228	0.887	1.656
Life Insurance	0.983	0.090	0.856	0.821	1.178
Extra IADL Limitation	1.406	0.106	0.000	1.213	1.629
Extra ADL Limitation	1.073	0.058	0.194	0.965	1.193
Any Nursing Home Visits previous 2 years	0.997	0.173	0.985	0.709	1.402
One Living Child, reference none	1.063	0.210	0.757	0.722	1.565
Two Living Children, reference none	1.293	0.225	0.139	0.920	1.819
Three or more Living Children, reference none	1.274	0.202	0.127	0.934	1.740
Living Near Friends	1.174	0.106	0.075	0.984	1.401
Currently Receiving Family Care	2.651	0.396	0.000	1.978	3.554
Help with chores/yardwork*	1.496	0.151	0.000	1.227	1.823
Constant	0.023	0.010	0.000	0.010	0.054

SOURCE [Authors’ analysis of data from the Health and Retirement Study, 2002-2018.]

NOTES [*Receive any help with yard work/house chores (not mentioned in ADL/IADL help) because of a health problem. N = 2,706; Pseudo R^2 = 0.173. Highlighted rows indicate variables with significant effects (p-value < 0.05)].
